# Metacognition moderates the effects of distraction on cognition

**DOI:** 10.3389/fpsyg.2015.00106

**Published:** 2015-02-09

**Authors:** Annelies Vredeveldt, Timothy J. Hollins

**Affiliations:** ^1^Department of Criminal Law and Criminology, VU University AmsterdamAmsterdam, Netherlands; ^2^School of Psychology, University of PlymouthPlymouth, UK

**Keywords:** distraction, cognition, metacognition, recognition, confidence

The work in the Research Topic illustrates the growing interest in the effects of distraction upon cognitive performance, in particular memory. In their excellent article, Beaman et al. (2014) point out that the majority of previous work on this topic has used memory tasks that allow little opportunity for the participants to demonstrate metacognitive monitoring or control of their performance. Given the myriad demonstrations of the influences of metacognitive monitoring and control on memory performance, this is a clear omission. For example, students studying for an exam may judge their own degree of learning (monitoring) and decide whether or not to study further (control). Similarly, eyewitnesses may evaluate their confidence in their memory (monitoring) and decide whether or not to make a positive identification decision (control). The paper sets out to investigate (1) whether distraction influences metacognition, as well as memory itself and (2) whether metacognition contributes to memory impairments.

Beaman and colleagues investigate distraction in the context of Koriat and Goldsmith's (1996) memory-control framework. This framework proposes that memory output in response to a cue is the result of three steps: generation of a best candidate answer, monitoring the quality of that answer (measured by confidence), and an output decision based upon comparing the monitored quality with output goals (measured by looking at the propensity to withhold answers). By comparing performance on tasks that allow withholding of answers (free-report) with performance on tasks that do not allow such control (forced-report), Koriat and Goldsmith were able to determine the trade-off between memory quantity and memory accuracy. Beaman and colleagues examined the effects of distraction on all aspects of the framework, by looking at: (a) the number of correct answers available (reflected in forced-report measures), (b) subjective assessments of the accuracy of candidate answers (reflected in confidence measures), and (c) the threshold at which participants are willing to report an answer (reflected in free-report and confidence measures).

In line with established effects in the literature, Beaman and colleagues show that distraction impacted upon retrieval of candidate answers (e.g., Glenberg et al., 1998; Vredeveldt et al., 2011; Perfect et al., 2012; Rae and Perfect, 2014). This is reassuring, but not new. The strength of the paper lies in the examination of metacognitive components of performance, which is both novel and thorough. Beaman and colleagues found that distraction impacted upon some, but not all metacognitive measures. Under distraction, participants were less able to distinguish their correct from incorrect answers (resolution). Whilst distraction did not affect the accuracy of answers volunteered in free report, it did result in fewer correct answers being volunteered. An intriguing aspect of performance was that distraction caused people to withhold answers more often, but did not change report threshold (as measured by confidence). Further detailed analysis demonstrated that this pattern arose because distraction lowered confidence in correct answers. As a result, participants found it harder to distinguish correct from incorrect answers and said “don't know” more often, despite maintaining the same criterion for outputting an answer.

Beaman and colleagues' examination of the impact of distraction on different metacognitive indices is both elegant and informative. It opens a fruitful avenue of research for others to follow, with clear theoretical and practical relevance. Four important issues spring to mind, but others will no doubt be inspired to take a different route. The authors themselves note that one issue will be to disentangle the effects of distraction on metacognition during encoding and retrieval, because the methodology used had distraction during both phases, but only measured metacognitive indices during retrieval. Disentangling these effects would address clear applied questions, for example: does distraction impair the ability to judge the degree of learning (cf. (Banbury and Berry, 1998; Hygge et al., 2003)), or the appropriate allocation of study time?

A related issue will be to investigate the effects of distraction on metacognition when memory quality varies. Many factors can impair the quality of the memory trace, for example, distraction or low attention during encoding, or long delays between encoding and retrieval. This raises the possibility that the metacognitive changes observed by Beaman and colleagues were not due to distraction during retrieval, but were the result of having to monitor lower-quality memory traces (due to distraction during encoding). This argument mirrors exactly the debate in another domain: the accuracy of feeling-of-knowing (FOK) judgements in younger and older adults. Whilst there is general agreement that older adults' episodic FOK judgements are less accurate, some have attributed this to poor monitoring at retrieval (Souchay et al., 2007), whilst others have attributed it to poor encoding (Perfect and Stollery, 1993; Hertzog et al., 2010).

The third issue concerns the nature of distraction: the present paper uses semantically meaningful, verbal distraction that is similar to the to-be-remembered material. It is unknown to what extent other forms of distraction impair metacognitive monitoring and control. Meaningless distraction during retrieval, such as moving shapes (Perfect et al., 2012), white noise (Perfect et al., 2011), or street noise (Vredeveldt and Penrod, 2013), has been found to impact memory performance, but these studies have lacked the metacognitive approach developed here (but cf. Vredeveldt and Sauer, 2015).

The final issue that remains unexplored is the impact of distraction upon sensitivity to output goals. Beaman and colleagues encouraged participants to maximize accuracy in free report, but there was no systematic variation in rewards and penalties associated with correct and incorrect responses. Koriat and Goldsmith (1994) found that people adjust their report threshold when the penalty for error is a small financial penalty, or the loss of all accrued rewards. Distraction could affect this strategic adjustment of report threshold.

In summary, Beaman and colleagues provide a stimulating approach to the examination of effects of distraction on performance, reminding us that memory retrieval in humans is the result of a subtle interplay between basic cognitive processes and metacognitive monitoring and control of those processes. They demonstrate that metacognitive impairments resulting from distraction can impair memory performance, and they offer a broad and sophisticated array of metacognitive measures with which to explore these issues. Our hope is that this work stimulates others to follow.

## Conflict of interest statement

The authors declare that the research was conducted in the absence of any commercial or financial relationships that could be construed as a potential conflict of interest.
